# Stereotactic Body Radiation Therapy for Pulmonary Oligometastases Arising from Non-lung Primaries in Patients Without Extrapulmonary Disease

**DOI:** 10.7759/cureus.2167

**Published:** 2018-02-07

**Authors:** Michael J Dohopolski, Zachary Horne, David Clump, Steven A Burton, Dwight E Heron

**Affiliations:** 1 Department of Radiation Oncology, UPMC Hillman Cancer Center; 2 Department of Radiation Oncology, University of Pittsburgh Cancer Institute, UPMC

**Keywords:** oligometastases, lung, pulmonary, local control, sbrt

## Abstract

Purpose

Stereotactic body radiation therapy (SBRT) is increasingly used in the management of patients with oligometastatic cancers and is under prospective evaluation by the Radiation Therapy Oncology Group (RTOG). Here we report outcomes from a high-volume institution of patients treated with SBRT for pulmonary oligometastases.

Materials and methods

We conducted a retrospective review of 105 patients who had one to five pulmonary oligometastases (185 lesions) without extrapulmonary disease treated with SBRT from 2002-2014. Target failure-free survival (TFFS), progression-free survival (PFS), and overall survival (OS) were calculated. Univariate and multivariate Cox regression analyses were performed on factors predictive of outcomes.

Results

The median age at first SBRT was 68 years and the median follow-up was 29.5 months. The median time from initial diagnosis of primary to SBRT was 42.7 months; 14.3% had synchronous oligometastases and 76.7% had one to two pulmonary lesions at first SBRT. The distribution of primaries was as follows: 36.2% colorectal, 16.2% head/neck, 9.5% genitourinary, 9.5% sarcoma, 7.6% gynecologic, 6.7% other, 5.7% breast, 5% melanoma, and 4% esophageal. The median lesion size was 1.6 cm and the most common regimen was 60 Gy in three fractions (range: 12-60 Gy in one to five fractions).

TFFS was 94.4% and 90.8% at two and three years, respectively. Two and three year OS were 87.9% and 60.2%, respectively. Median PFS and OS were 16.2 and 45.3 months, respectively. In multivariate analysis, age at primary cancer diagnosis and biologically effective dose with an alpha-beta ratio of 10 (BED10) were identified as factors significantly affecting OS (p<0.05).

Conclusions

Comprehensive treatment of pulmonary oligometastases with SBRT in the absence of extrapulmonary disease results in excellent target control and modest survival outcomes.

## Introduction

The development of metastatic disease has traditionally been considered the end-stage in the natural history of malignancy. Yet, there are documented subsets of patients with limited pulmonary dissemination, described as being in an oligometastatic state, that experience an overall survival (OS) benefit from local therapies, such as surgical metastasectomy [1]. Not all patients are willing or able to undergo a surgical resection, however. Stereotactic body radiation therapy (SBRT), proven to be tolerable and efficacious in treating patients with medically-inoperable early-stage non-small cell lung cancer (NSCLC), has emerged as an option for the treatment of patients with pulmonary oligometastatic disease who are unable to tolerate or who do not wish to undergo surgical resection [2-6]. Data illustrating the benefits of SBRT for patients with pulmonary oligometastatic disease have been limited to small prospective and/or heterogeneous retrospective studies [7, 8]. Moreover, identifying patients who have the highest likelihood of prolonged remission or cure is still elusive. 

In 2010, a systematic review summarized the outcomes from 19 institutions (739 target lesions) using single and/or hypofractionated (33 Gy/six fractions to 60 Gy/three fractions) SBRT for treating pulmonary oligometastases. The therapies were well-tolerated and demonstrated high rates of local control (LC, two year 67-95%) [8]. However, conclusions concerning the optimal treatment regimen were limited due to treatment heterogeneity. More recently in 2016, a multi-institutional retrospective analysis was performed on 700 patients, which demonstrated similar rates of LC (two year 81%). While treatments were again heterogeneous, they were still able to note promising effects on OS and identify several prognostic factors—performance status, tumor size, number of metastases, and interval between primary tumor diagnosis to SBRT [5]. 

The purpose of this retrospective single-institutional study is to further assess the efficacy, patterns of failure, and outcomes of SBRT in the treatment of lung oligometastases arising from non-lung primary malignancies and their relationship with baseline characteristics such as histology, size, and treatment characteristics such as dose fractionation.

## Materials and methods

Patient selection

We performed a retrospective analysis on 105 patients with 185 pulmonary lesions treated from 2002 to 2014 with SBRT. Patients who were ≥18 years old with one to five total lung-only metastases from non-lung primaries were included in the study. Metastatic disease was clinically diagnosed based on computed tomography (CT) and/or fluorodeoxyglucose (FDG)-positron emission tomography (PET) imaging, with or without biopsy as deemed appropriate by a multidisciplinary team consisting of a radiation oncologist, medical oncologist, and thoracic surgeon. Patients were then seen in a multidisciplinary clinic where treatment recommendations were provided. Dose prescription was determined on a per-patient basis and was dependent on surrounding critical structures and prior therapies.

Definition of variables

Target failure (TF) consisted of progression of the treated lesion. In-lobe failure (LF) consisted of any failure in the same lobe as the treated lesion excluding the treated lesion. Regional failure (RF) was defined as failure in the ipsilateral lung, hilar, and mediastinal lymph nodes, and distant metastases (DM) consisted of any new contralateral nodal or pulmonary parenchymal disease or extrapulmonary disease. Progression-free survival (PFS) was measured from the time of SBRT until respective failure or death. Primary tumor origin was categorized as esophageal, colorectal, gynecologic (GYN), head and neck, sarcoma, genitourinary (GU), breast, melanoma, and other. The category “other” included adrenal, pancreas, liver, and spine (ependymoma) because of the small number of cases. Therapies prior to SBRT included chemo/hormonal therapy, radiotherapy, radiofrequency ablation, and/or surgery.

OS was measured from the start of SBRT to death by any cause or date of last contact. Because some patients had multiple lesions treated with SBRT, OS was calculated from the start of the initial course of SBRT per-patient (n=105). TF-free survival (TFFS), in-lobe failure-free survival (LFFS), regional failure-free survival (RFFS), distant metastasis-free survival (DMFS), and PFS were calculated using total lesion treated data (n=185). Patients were censored if they were still alive at date of last contact.

Simulation and contouring

While immobilized in custom BodyFixTM, patients underwent CT simulation with their arms raised above their heads. The real-time position management (RPM) system was used when thin-sliced 4D-CT scans with intravenous contrast were performed. On imaging, the visible tumor—defined as the gross tumor volume (GTV)— including spiculations, was synchronized with fiducial movement when patients were treated with CyberKnife™ using Synchrony Respiratory Tracking System (Accuray Inc., Sunnyvale, CA). Varian Real-Time Position Management System (Varian Medical Systems, Palo Alto, CA) was used when patients were treated on Trilogy™ and Trubeam™ machines. Respiratory gating was used when > 0.5 cm motion was noted. The pencil beam planning algorithm was used on CyberKnife™ machines and the anisotropic analytical algorithm (AAA) was used on Trilogy™ and Trubeam™ machines. The normal lung, heart, spinal cord, esophagus, and brachial plexus were included as normal structures in the planning.

Contouring was completed on the CT50. An expansion of 3-5 mm was added to the GTV (forming the planning target volume—PTV) to address motion and daily set up error. The goal, utilizing the dose-volume histograms, was to deliver 95% of the prescription dose to the PTV while also limiting dose to surrounding critical structures. Adjustments to the regimens were made according to critical structure constraints. The patients were treated every other day.

Statistical analysis

Data analyses were performed using IBM SPSS version 24.0 (IBM Corp., Armonk, NY). All factors potentially predictive for outcomes were identified using univariate Cox regression analysis. Factors with p < 0.10 on univariate analysis were included in a Cox multivariate regression model which used backward conditional selection. Survival statistics were calculated using the Kaplan-Meier method with log-rank test. A p value < 0.05 was used to establish statistical significance.

## Results

Baseline characteristics

Patient demographics and baseline characteristics are given in Table 1. The median age of cancer diagnosis was 63 years (interquartile range, IQR: 52-71 years old). The median age at diagnosis of any metastases was 65 years (IQR: 58-73 years old), and the median age at first SBRT was 68 years (IQR: 59-75 years old). The median follow-up from SBRT was 29.5 months (IQR: 14.5-47.4 months) using per-patient data (n=105). Forty-three (42.7) months was the median time from initial diagnosis of malignancy to SBRT. Gastrointestinal primaries were the most common (36.2%) in our cohort. Adenocarcinoma represented approximately half (51.1%) of the primary histologies. The majority (85.7%) of patients were diagnosed with metachronous metastatic disease. The median lesion size was 1.6 cm (IQR: 1.0-2.3 cm).

**Table 1:** Baseline cohort characteristics stereotactic body radiation therapy (SBRT), genitourinary (GU), gynecologic (GYN)

Baseline Characteristics	Number of patients (%)
Sociodemographic Factors	
Age at Diagnosis of Primary Malignancy	
≤ 50	21 (20.0)
51-69	55 (52.4)
≥ 70	29 (27.6)
Age at SBRT	
≤ 50	9 (8.6)
51-69	48 (45.7)
≥ 70	48 (45.7)
Sex	
Male	54 (51.4)
Female	51 (48.6)
Race	
Caucasian	99 (94.3)
African American	4 (3.8)
Asian	2 (1.9)
Pathological Factors	
Primary Origin	
Colorectal	38 (36.2)
Head/Neck	17 (16.2)
GU	10 (9.5)
Sarcoma	10 (9.5)
GYN	8 (7.6)
Other	7 (6.7)
Breast	6 (5.7)
Melanoma	5 (4.8)
Esophagus	4 (3.6)
Histology	
Adenocarcinoma	54 (51.4)
Squamous Cell Carcinoma	13 (12.4)
Sarcoma	16 (15.2)
Other	22 (21.0)
Synchronous or Metachronous	
Synchronous	15 (14.3)
Metachronous	90 (85.7)
Location of First Metastasis	
Lung	111 (60)
Non-lung Viscera	20 (10.8)
Nodal	39 (21.1)
Bone	1 (0.5)
Multiple Sites	14 (7.6)
Therapeutic Factors (Total lesions treated)	
Prior Treatment	
Chemo/Hormonal Therapy	81 (43.8)
Radiation Therapy	2 (1.1)
Surgery	18 (9.7)
Multiple Lines	64 (34.6)
None	20 (10.8)
Post SBRT Chemotherapy	
Yes	76 (41.1)
No	109 (58.9)
SBRT Characteristics (total lesions treated)	
Dose (Gy)	
60 in three fractions	73 (39.5)
48 in four fractions	65 (35.1)
54 in three fractions	22 (11.9)
others	25 (13.5)
Number of lesions at SBRT	
One	95 (51.4)
Two	47 (25.3)
Three	21 (11.4)
Four to five	22 (11.9)

Treatment

At the time of the first SBRT, 89.5% of patients had undergone prior therapies (systematic/chemotherapy, surgery, radiation, or a combination of modalities) for metastatic disease. A significant majority of these patients (88.6%) had one to two lesions treated at the time of SBRT. The most common SBRT regimen was 60 Gy in three fractions (39.5%). The second and third most common regimens were 48 Gy in four fractions (35.1%) and 54 Gy in three fractions (13.5%), respectively (range: 12-60 Gy in one to five fractions). Eighty-seven percent (87%) of the delivered treatment regimens had a biologically effective dose with alpha-beta ratio of 10 (BED10) ≥100Gy.

Disease outcomes

**Figure 1:**
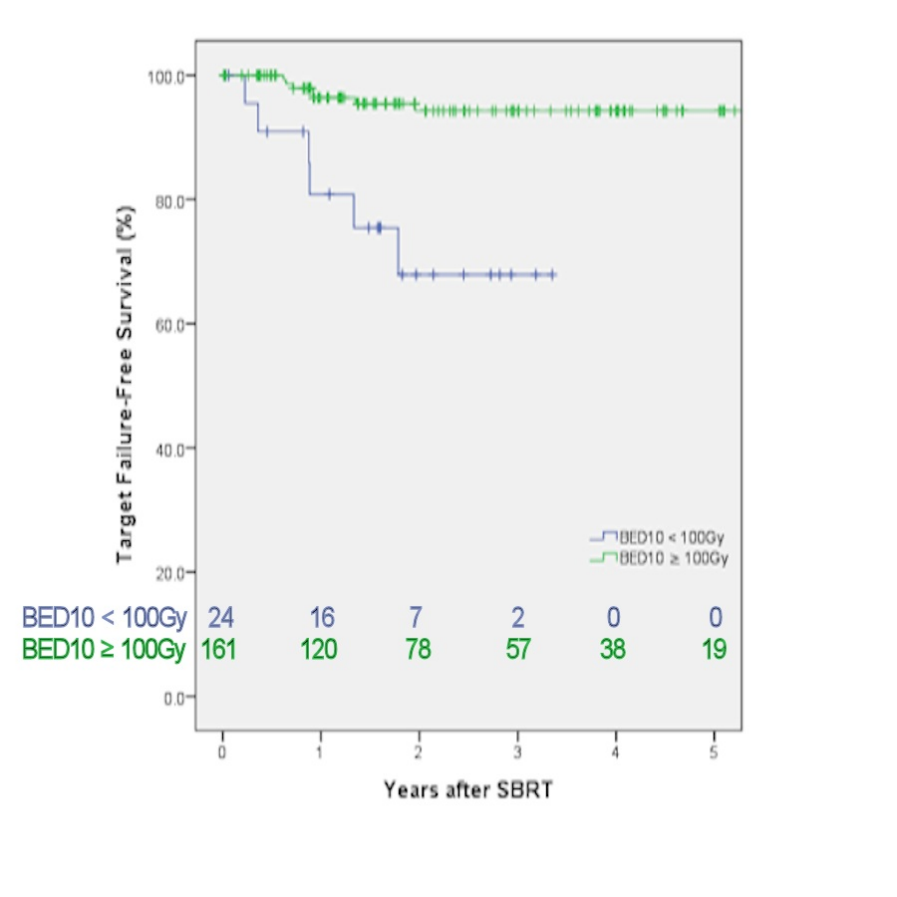
Improved target failure-free survival with lesions treated to BED10 ≥100 Gy stereotactic body radiation therapy (SBRT), biologically effective dose with alpha-beta ratio of 10 (BED10)

TFFS, LFFS, RFFS, DMFS, PFS, and OS at two and three years can be seen in Table 2. Two and three year TFFS were 94.4% and 90.8%, respectively. Lesions treated to a BED10 ≥100Gy (Figure 1) or tumors ≤1.6 cm had significantly greater target control (p=0.001). Fewer target failures were noted when more than one lesion was treated (p=0.003); however, more treated lesions were associated with smaller tumor size per lesion (p<0.05). Improved LFFS was noted with lesions ≤1.6 cm (p=0.03) and lesions treated to a BED10 ≥100Gy (p=0.03). RFFS was improved with lesions ≤1.6 cm (p<0.001), BED10 ≥100Gy (p<0.001), and treating more than three lesions (p<0.05). Improved DMFS was associated with treating more than three lesions (p=0.04). Treating a lesion to a BED10 ≥100 Gy did not significantly alter DMFS. Treating tumors ≤1.6 cm, treating more than one lesion, and treating to BED10 ≥100 Gy were all associated with better PFS (p<0.05). Trends toward greater OS were seen with tumors ≤1.6 cm (p=0.10), while no benefit in OS was seen in lesions treated to BED10 ≥100 Gy (p=0.20). Even more stringent criteria were necessary to observe improvements in overall survival. Statistically significant improvements in OS were observed when tumors were ≤1.4 cm and treated to a BED10 ≥120 Gy (p<0.05); smaller lesions were associated with greater BED10 (p<0.05).

Of the 48 patients who failed distantly, 19 failed in the contralateral lung or node and 29 failed extrapulmonary—some experienced both contralateral lung and pulmonary failure.

**Table 2:** Failure patterns and progression-free survival

Failure after SBRT	Number of treated lesions (%)
Target-lesion	13 (7.0)
In-lobe	41 (22.2)
Regional	44 (23.8)
Distant	48 (25.9)
Progression Free Survival (from all lesion data)	(%)
Target failure-free survival	
Two year	94.4
Three year	90.8
In-lobe failure-free survival	
Two year	82.4
Three year	72.9
Regional failure-free survival	
Two year	81.1
Three year	70.3
Distant metastases-free survival	
Two year	81.8
Three year	67.9
Progression-free survival	
Two year	61.1
Three year	43.0
Five year	41.6
Overall Survival	(%)
Two year	87.9
Three year	60.2
Five year	43.0

Univariate and multivariate analysis

Multivariate and univariate analyses were performed on factors influencing TFFS, LFFS, RFFS, DMFS, PFS, and OS. Hazard ratios (HR) and 95% confidence intervals (CI) for these analyses can be found in Table 3 (below) and Table 4(supplemental, see Appendix).

**Table 3:** Multivariate analyses for target failure-free survival, in-lobe failure-free survival, regional failure-free survival, distant metastasis failure-free survival, progression-free survival, and overall survival stereotactic body radiation therapy (SBRT), genitourinary (GU), gynecologic (GYN), biologically effective dose with alpha-beta ratio of 10 (BED10)

Multivariate Analysis	Overall survival		Target failure-free survival	
Factors	HR (95% CI)	P Value	HR (95% CI)	P Value
Age				
At SBRT	-	-	-	-
At diagnosis	1.02 (1.01-1.04)	0.01	-	-
Tumor Size	1.30 (0.97-1.75)	0.08	1.87 (1.10-3.12)	0.02
Primary Origin				
Breast	Reference		Reference	
Esophagus	3.56 (0.84-15.1)	0.09	1.35 (0.13-13.7)	0.80
GYN	1.28 (0.29-5.57)	0.75	0.05 (0.00-1.20)	0.07
Head/Neck	1.84 (0.51-6.58)	0.35	*	0.97
Sarcoma	3.63 (0.87-15.2)	0.08	0.63 (0.05-7.92)	0.72
GU	0.69 (0.17-2.91)	0.62	1.32 (0.11-16.0)	0.83
Colorectal	0.86 (0.26-2.85)	0.80	1.17 (0.28-7.24)	0.68
Melanoma	1.33 (0.28-6.29)	0.72	*	0.99
Other	0.57 (0.10-3.40)	0.54	*	0.99
Number lesions at SBRT	-	-	0.26 (0.04-1.80)	0.17
BED10	0.99 (0.985-0.999)	0.02	0.98 (0.96-0.997)	0.02
Multivariate Analysis	In-lobe failure-free survival		Regional failure-free survival	
Factors	HR (95% CI)	P Value	HR (95% CI)	P Value
Tumor Size	1.41 (1.17-1.70)	<0.001	-	-
Number lesions at SBRT	-	-	0.49 (0.31-0.76)	0.001
BED10	-	-	0.99 (0.98-0.997)	0.003
Time to 1^st^ SBRT	-	-	0.99 (0.985-0.999)	0.02
Post SBRT Chemotherapy	2.08 (1.10-3.91)	0.02	-	-
Multivariate Analysis	Distant metastasis failure-free survival		Progression-free survival	
Factors	HR (95% CI)	P Value	HR (95% CI)	P Value
Number lesions at SBRT	0.56 (0.38-0.82)	0.003	0.56 (0.44-0.72)	<0.001
Post SBRT Chemotherapy	2.65 (1.49-4.71)	<0.001	2.61 (1.72-3.98)	<0.001

## Discussion

Much like surgery, one of the goals of SBRT is to eliminate local disease. This is especially important in the setting of oligometastatic disease as local control limits native tissue destruction, and in theory, helps prevent further systemic spread from these oligometastatic foci. Target failure within our cohort was quite low (three year target progression rate of 9.2%) and, in many cases, comparable to surgical resection [9]. We identified several factors that affected TFFS including BED10 and tumor size which have been noted in several other studies [5, 10].

In-lobe, regional, and distant progression were more common within our cohort compared to target progression (two year LFFS/RFFS/DMFS vs TFFS were 82.4%/81.1%/81.8% vs 94.4%, respectively). This is expected as SBRT principally focuses on gross tumor; moreover, by definition, these individuals already have disseminated disease and likely have a greater propensity for distant spread. Multivariate analyses identified several positive prognostic factors that similarly affected LFFS, RFFS, and DMFS. One predominant variable included treating more versus fewer lesions at SBRT; this finding was initially counterintuitive until further analyses demonstrated that patients treated for multiple lesions typically had smaller size lesions. Our findings support the notion that total volume of disease is a poorer prognostic factor than the total number of lesions; however, we acknowledge that the decreased rates of progression, excluding target progression, observed treating smaller lesion might be a manifestation of lead-time bias and not an actual benefit from treatment. An alternative explanation explored was if treating more lesions increased the likelihood of inducing possible abscopal effects—SBRT promoting an immune response that may eradicate micrometastases in non-irradiated areas [11]. The literature suggests, though, that induction of the abscopal effect is more a function of biologically effective dose, and BED10 within our cohort was not shown on multivariate analyses to be a positive prognostic marker for RFFS, DMFS, or PFS making the abscopal explanation possibly less likely [12]. We await the results from the NRG trials, BR001 and BR002, for further evidence in treating oligometastases with radiation therapy.

Oligometastases, as previously mentioned, have been hypothesized to represent a state of limited systemic disease from which cure or prolonged remission may be achieved. One of the first studies supporting this theory was a surgical series evaluating over 5,200 patients undergoing pulmonary metastasectomy that showed five and 15-year survival rates of 36% and 22%, respectively. Evidence is now accumulating that suggests SBRT to have similar OS (five year OS 22-38%) rates to metastasectomy [13, 14]. At the moment, however, it is difficult to adequately assess the survival outcomes of patients with pulmonary metastases treated with SBRT, especially compared to surgical resection, as there are a paucity of randomized clinical trials addressing this topic and the data that is available from phase I/II trials encompass a diverse patient population [7, 15]. There has been one retrospective series that compared SBRT to metastasectomy that illustrated equivalency; however, they focused on patients with osteosarcoma [16]. Further complicating appropriate comparisons are the patient populations themselves, as patients receiving radiation frequently are not surgical candidates due to significant comorbidities. These comorbidities not only limit their ability to tolerate other therapies such as systemic treatment but in some instances, contribute to their cause of death. Our data further support the similarities in outcomes between metastasectomy and SBRT as our five year OS (43.0%) is similar to surgical intervention.

We also observed several factors that influenced OS. Factors significant on univariate analysis can be found in Table 4 but those that were still significant on multivariate analysis included age at primary cancer diagnosis and BED10. The benefit of higher BED10 in OS was likely a reflection tumor location allowing for more optimal dose delivery thus improving local control. Factors trending toward significance were disease from esophageal (HR 3.56, p=0.09) and sarcoma primaries (HR 3.63, p=0.08) and tumor size (1.30, p=0.08). Several of these factors have already been discussed in the literature [5, 17]. One of the worse prognostic factors trending towards significance within our cohort was pulmonary oligometastases from esophageal primaries when compared to breast primaries. To our knowledge, we are unaware of other studies that have noted this potential correlation. However, this might be attributed to the overall aggressive nature of esophageal cancer. Lacking similar correlations in target failure makes it less likely that the issue is a response to radiation therapy. Studies to date have typically identified metastases from colorectal primaries or melanoma to be associated with poorer OS [5, 13, 18].

Limitations of this study include those prominent to retrospective reviews: ascertainment bias, incomplete data, and loss of follow-up. We also did not collect toxicity data; however, several other studies have noted limited events of grade three and greater toxicity [5, 7, 8, 19]. Location (central versus peripheral) and large tumor size have been factors associated with increased toxicity. An early study evaluating treatment of centrally located lesions (within two cm of the trachea and proximal bronchial tree) with SBRT even noted grade five toxicities [20]. Efforts are now made to decrease the incidence of such events either by changing the dose or number of fractionations [5, 21]. Performance status was also not recorded—a factor associated with decreased OS and local control [5, 22]. Despite these limitations, this is the largest series looking at treating all lung-only disease with SBRT in the absence of extrapulmonary disease with modest median follow-up.

## Conclusions

Treating all pulmonary oligometastases originating from non-lung primaries with SBRT in the absence of extrapulmonary disease results in excellent target control especially for smaller lesions. It is likely target control is similar to that achieved by surgical resection but validation through prospective studies are needed. In the interim, patients appear to benefit from this highly-focal dose of SBRT for pulmonary oligometastatic disease.

## Appendices

**Table 4:** Univariate analyses for target failure-free survival, in-lobe failure-free survival, regional failure-free survival, distant metastasis failure-free survival, overall progression-free survival, and overall survival stereotactic body radiation therapy (SBRT), genitourinary (GU), gynecologic (GYN), biologically effective dose with alpha-beta ratio of 10 (BED10), target failure-free survival (TFFS), in-lobe failure-free survival (LFFS), regional failure-free survival (RFFS), distant metastasis failure-free survival (DMFS), progression free survival (PFS)

Univariate Analysis	Overall survival		TFFS	
Factors	HR (95% CI)	P Value	HR (95% CI)	P Value
Race				
Caucasian	Reference		Reference	
African American	*	0.98	0.04 (0.00-437)	0.51
Age				
At SBRT	1.02 (0.99-1.04)	0.18	1.01 (0.97-1.06)	0.51
At diagnosis	1.02 (1.00-1.05)	0.04	1.00 (0.97-1.05)	0.84
Prior treatments				
None	Reference		Reference	
Had Prior	0.57 (0.25-1.27)	0.17	1.18 (0.15-9.15)	0.87
Synch/Metachronous				
Synchronous	Reference		Reference	
Metachronous	1.08 (0.48-2.40)	0.86	1.56 (0.20-12.0)	0.67
Tumor Size	1.52 (1.21-1.92)	<0.001	1.97 (1.49-2.61)	<0.001
Primary Origin				
Breast	Reference		Reference	
Esophagus	3.39 (0.83-13.8)	0.09	0.46 (0.05-4.51)	0.51
GYN	0.88 (0.22-3.57)	0.85	0.25 (0.03-2.44)	0.24
Head/Neck	1.27 (0.39-4.15)	0.69	*	0.97
Sarcoma	2.20 (0.61-7.91)	0.23	0.46 (0.08-2.78)	0.40
GU	0.59 (0.15-2.37)	0.46	0.31 (0.02-1.98)	0.17
Colorectal	0.82 (0.27-2.45)	0.72	0.27 (0.07-1.18)	0.08
Melanoma	0.92 (0.20-4.16)	0.91	*	0.99
Other	0.50 (0.09-2.75)	0.43	*	0.98
Histology				
Adenocarcinoma	Reference		Reference	
Squamous Cell Carcinoma	1.22 (0.53-2.84)	0.64	*	0.98
Sarcoma	1.29 (0.60-2.78)	0.51	1.64 (0.49-5.45)	0.42
Other	0.73 (0.35-1.54)	0.41	0.34 (0.04-2.75)	0.31
Number lesions at SBRT	1.14 (0.84-1.55)	0.41	0.14 (0.02-0.96)	0.045
BED10	0.99 (0.985-0.997)	0.002	0.97 (0.96-0.99)	<0.001
Time to 1^st^ SBRT	0.995 (0.99-1.00)	0.07	1.00 (0.99-1.01)	0.65
Post SBRT Chemotherapy	1.33 (0.76-2.31)	0.32	0.91 (0.30-2.80)	0.87
Univariate Analysis	LFFS		RFFS	
Factors	HR (95% CI)	P Value	HR (95% CI)	P Value
Race				
Caucasian	Reference		Reference	
African American	0.30 (0.04-2.16)	0.23	0.66 (0.16-2.27)	0.56
Age				
At SBRT	0.99 (0.97-1.02)	0.56	1.02 (0.99-1.04)	0.18
At diagnosis	1.00 (0.97-1.02)	0.71	1.02 (1.00-1.05)	0.049
Prior treatments				
None	Reference		Reference	
Had Prior	2.00 (0.48-8.29)	0.34	0.97 (0.34-2.70)	0.95
Synch/Metachronous				
Synchronous	Reference		Reference	
Metachronous	0.78 (0.45-2.90)	0.78	0.97 (0.38-2.45)	0.94
Tumor Size	1.47 (1.21-1.79)	<0.001	1.33 (1.09-1.61)	0.004
Primary Origin				
Breast	Reference		Reference	
Esophagus	2.45 (0.54-11.0)	0.24	1.21 (0.27-5.43)	0.80
GYN	0.25 (0.03-2.41)	0.23	0.65 (0.15-2.92)	0.58
Head/Neck	0.80 (0.20-3.22)	0.76	0.75 (0.23-2.50)	0.64
Sarcoma	0.95 (0.21-4.26)	0.95	0.93 (0.25-3.50)	0.92
GU	0.63 (0.13-3.12)	0.57	0.31 (0.06-1.69)	0.17
Colorectal	1.11 (0.33-3.77)	0.87	0.52 (0.17-1.62)	0.26
Melanoma	0.50 (0.05-4.79)	0.55	0.76 (0.14-4.14)	0.75
Other	0.23 (0.02-2.21)	0.20	1.04 (0.28-3.88)	0.95
Histology				
Adenocarcinoma	Reference		Reference	
Squamous Cell Carcinoma	0.72 (0.28-1.90)	0.52	0.96 (0.42-2.20)	0.92
Sarcoma	0.70 (0.29-1.70)	0.43	0.69 (0.29-1.68)	0.42
Other	0.41 (0.14-1.18)	0.097	0.38 (0.13-1.08)	0.07
Number lesions at SBRT	0.86 (0.64-1.15)	0.30	0.50 (0.32-0.77)	0.002
BED10	0.994 (0.99-1.002)	0.12	0.99 (0.98-1.00)	0.005
Time to 1^st^ SBRT	0.998 (0.99-1.00)	0.55	0.99 (0.986-1.00)	0.049
Post SBRT Chemotherapy	2.25 (1.21-4.19)	0.01	1.99 (1.04-3.43)	0.04
Univariate Analysis	DMFS		PFS	
Factors	HR (95% CI)	P Value	HR (95% CI)	P Value
Race				
Caucasian	Reference		Reference	
African American	0.88 (0.27-2.84)	0.83	0.58 (0.21-1.58)	0.29
Age				
At SBRT	1.01 (0.99-1.03)	0.47	1.00 (0.98-1.02)	0.94
At diagnosis	1.01 (0.99-1.02)	0.39	1.00 (0.99-1.02)	0.62
Prior treatments				
None	Reference		Reference	
Had Prior	1.39 (0.43-4.49)	0.59	1.08 (0.62-2.24)	0.83
Synch/Metachronous				
Synchronous	Reference		Reference	
Metachronous	0.59 (0.28-1.27)	0.18	0.69 (0.38-1.24)	0.21
Tumor Size	1.17 (0.93-1.47)	0.18	1.31 (1.14-1.52)	<0.001
Primary Origin				
Breast	Reference			
Esophagus	1.33 (0.35-5.03)	0.67	0.76 (0.27-2.15)	0.61
GYN	0.78 (0.21-2.92)	0.71	0.55 (0.21-1.42)	0.22
Head/Neck	0.29 (0.08-1.07)	0.06	0.36 (0.15-0.85)	0.02
Sarcoma	1.47 (0.47-4.59)	0.51	0.64 (0.26-1.53)	0.31
GU	0.44 (0.12-1.65)	0.22	0.21 (0.07-0.64)	0.01
Colorectal	0.39 (0.14-1.11)	0.08	0.39 (0.18-0.82)	0.01
Melanoma	1.30 (0.31-5.46)	0.72	0.79 (0.26-2.36)	0.67
Other	0.62 (0.17-2.30)	0.47	0.38 (0.13-1.06)	0.06
Histology				
Adenocarcinoma	Reference		Reference	
Squamous Cell Carcinoma	1.00 (0.56-1.82)	0.99	0.73 (0.38-1.40)	0.35
Sarcoma	1.37 (0.87-2.15)	0.17	0.94 (0.54-1.62)	0.81
Other	1.16 (0.65-2.06)	0.62	0.65 (0.35-1.20)	0.17
Number lesions at SBRT	0.56 (0.38-0.82)	0.003	0.60 (0.47-0.77)	<0.001
BED10	1.00 (0.99-1.01)	0.80	0.996 (0.99-1.00)	0.08
Time to 1^st^ SBRT	0.998 (0.99-1.00)	0.48	0.998 (0.994-1.00)	0.20
Post SBRT Chemotherapy	2.59 (1.46-4.61)	0.001	2.19 (1.44-3.31)	<0.001
